# Comparative Analysis of Extracellular Vesicles in Patients with Severe and Mild Myalgic Encephalomyelitis/Chronic Fatigue Syndrome

**DOI:** 10.3389/fimmu.2022.841910

**Published:** 2022-03-04

**Authors:** Hector Bonilla, Dylan Hampton, Erika G. Marques de Menezes, Xutao Deng, José G. Montoya, Jill Anderson, Philip J. Norris

**Affiliations:** ^1^ Department of Medicine, Stanford University, Palo Alto, CA, United States; ^2^ Vitalant Research Institute, San Francisco, CA, United States; ^3^ Department of Laboratory Medicine, University of California, San Francisco, San Francisco, CA, United States; ^4^ Department of Medicine, University of California, San Francisco, San Francisco, CA, United States

**Keywords:** extracellular vesicle (EV), ME/CFS, inflammation, cell signaling, soluble factors of immune system

## Abstract

Myalgic encephalomyelitis, or chronic fatigue syndrome (ME/CFS) is a serious disease whose cause has yet to be identified. Objective markers of the disease are also not well understood and would serve as important tools in diagnosis and management. One potential biomarker or transmitter of immune signals in ME/CFS is the extracellular vesicle (EV) compartment. These small, membrane bound particles have been shown to play a key role in intercellular signaling. Our laboratory has focused on methods of detection of EVS in clinical samples. In this study we explored whether the prevalence of EVs in the plasma of participants with mild or severe ME/CFS differed from the plasma of healthy control participants. By staining for multiple cell surface molecules, plasma EVs could be fingerprinted as to their cell of origin. Our study revealed a significant correlation between severe ME/CSF and levels of EVs bearing the B cell marker CD19 and the platelet marker CD41a, though these changes were not significant after correction for multiple comparisons. These findings point to potential dysregulation of B cell and platelet activation or homeostasis in ME/CFS, which warrants validation in a replication cohort and further exploration of potential mechanisms underlying the association.

## Introduction

Myalgic encephalomyelitis, or chronic fatigue syndrome (ME/CFS) is a debilitating disease that afflicts between 836,000 to 2.5 million Americans (1). Based on the Fukuda CDC case definition, the worldwide prevalence of ME/CFS is estimated at 0.89%, and women represent 1.5 to 2 times higher than the male population. Core symptoms include exertion intolerance, cognitive impairment, non-restorative sleep, and a reduced ability to engage in occupational, educational, social, and personal activities. Most patients who are ill for longer than 5 years never regain their pre-illness levels of health or functioning (1). ME/CFS patients have a lower quality of life scores than other illnesses such as stroke, chronic renal failure, lung, breast, or colon cancer (2). Persistent fatigue introduces social and financial hardships, as 80% of patients cannot work or attend school, while 25% are confined to their homes or beds. Until recently, the disease has been heavily stigmatized and incorrectly treated as psychogenic or misdiagnosed as depression (3). However, very little is known about the etiology of the illness, with no established diagnostic test or FDA-approved treatments.

Extracellular vesicles (EVs) are lipid bilayer particles released by the cell and represent a mechanism of intercellular communication found in multiple cell types and various body fluids (4). Their size varies from 40-120 nanometer (exosomes) to 100-1000 nanometer (microvesicles) (5, 6). The majority of EVs are less than 200 nm in size. EVs are cargo vesicles that carry proteins, nucleic acids, lipids, metabolites, and other cytosolic components (7–9). Proteins common in EVs have been used as markers in order to tag them, such as Alix, tetraspanins (CD63, CD81), and heat shock proteins (HSP70, 90) (10). Analysis of EVs has emerged as yielding potential markers for conditions such as cancer, cardiovascular disease (11), progression of Parkinson’s disease, and differentiation of neurodegenerative diseases such as Lewy body dementia vs. Alzheimer’s disease (5, 6, 12). Small studies in patients with ME/CFS indicate total EV counts were significantly higher in ME/CFS compared to healthy controls in two studies (7, 8), but not in two others (10, 11). We proposed a study to explore whether levels of EVs or EV phenotype are associated with ME/CFS and ME/CFS severity.

## Materials and Methods

### Study Subjects and Sample Collection

Samples were selected from the Stanford repository of 192 patients with ME/CFS, with a mean age of 49.9 years and 76.6% female. The Multidimensional Fatigue Inventory (MFI-20) measured the severity of the illness for the patients with ME/CFS (13). Twenty plasma samples were selected for EV analysis based on the extremes of clinical scores and sample availability, corresponding to 10 with mild (MFI score 51-75) and 10 with severe ME/CFS (MFI-20 score of 86-100). In addition, plasma samples were tested from 20 healthy control participants who were matched to the ME/CFS participants based on age and sex. The study participants provided written informed consent, and studies were approved by the Stanford University Institutional Review Board (protocol numbers 18068 and 18155). Participants were classified as cases if they met the 1994 CDC CFS case definition (14). Exclusion criteria included active or uncontrolled morbidities that would have interfered with the patient**’**s ability to participate in the study, particularly conditions or medications causing immunosuppression or immunodeficiency. The questionnaire was administered to each participant on the day of blood sample collection. Blood was collected in an EDTA tube and centrifuged within an hour of collection, at 1800 g for 15 minutes and cryopreserved at -70°C.

### EV Quantitation and Phenotyping

EVs were measured directly from cryopreserved plasma samples that were thawed and centrifuged at 4°C for 15 minutes. Samples were stained with pre-titrated volumes of the following fluorochrome conjugated monoclonal antibodies in four separate panels purchased from BioLegend except as noted below: CD3-FITC, CD4-PE, CD11b-PE-Cy7, CD14-APC, CD16-V450 (BD Biosciences), CD19-PE-Cy7 (BD Biosciences), CD40-FITC, CD41a-PerCP-Cy5.5, CD62P-FITC (BD Biosciences), CD63-APC, CD66b-PerCP-Cy5.5, CD154-APC, CD163-PE-Cy7, CD192-BV421, CD195-PE (BD Biosciences), CD200-PerCP-Cy5.5, microtubule associated protein-2 (MAP2)-AlexaFluor488 (BD Biosciences), glial fibrillary acidic protein (GFAP)-BV421, macrophage colony stimulating factor (M-CSF)-PE, and CX3CR1-BV421 (BD Biosciences). Details of volumes used, clones, isotype, and catalog numbers are listed in **Table S1**. Prior to staining, each antibody was filtered using a 0.22 µm centrifugal filter (Ultrafree MC-GV centrifugal filters, Millipore) to remove aggregates. One to 4 µl of titrated monoclonal antibodies was added to 10 μl of plasma and incubated at 4°C for 30 minutes. EVs were diluted 1:100 in buffered 0.22 µm-filtered PBS. Samples were acquired on an LSR II flow cytometer (BD). Samples were collected for 60 seconds, and EV counts were calculated using TruCount tubes (BD Biosciences), which allowed normalization of the flow rate using the known amount of fluorescent beads in the TruCount tube (15). EV gates were set using polystyrene beads ranging from 100 nm to 1 μm (Sperhotech and Megamix beads). Analysis was performed using FlowJo 10 software (BD Biosciences).

### Nanoparticle Tracking Analysis

Concentration and size distribution profile of the particles was evaluated using a Nanosight NS300 instrument (Malvern) configured with syringe pump, a 405 nm laser, and a high sensitivity scientific sCMOS camera. Five 60-second videos were recorded for each sample with camera level 15 and the detection threshold set at 5. Ten µL of EV samples were diluted 1:10,000 in PBS and filtered with a 0.22 µm pore filter membrane. Data were analyzed using NTA 3.3 software. The nanoparticle tracking analysis (NTA) measurements were performed on plasma aliquots that had undergone one additional freeze-thaw compared to those measured by flow cytometry.

### Statistical Analysis

EV data were log-transformed prior to analysis. Comparisons across the healthy control, mild, and severe groups were made by one-way ANOVA with a Tukey’s multiple comparison test. Analysis of covariance (ANCOVA) was performed to test for interaction between participant age and the relationship between EVs and disease state (R/stats package). Comparison of MFI-20 scores between mild and severe cases was performed using a t-test. Comparisons of gender across clinical groups were made using a Chi-square test. Analyses were performed using GraphPad Prism, version 9.1.2.

## Results

### Study Subjects

Cases were classified into tertiles for ME/CFS severity: MFI-20 scores from 51**–**75 were classified as a mild disease, scores from 76**–**85 as moderate disease, and scores from 86**–**100 as severe disease (16). Of note, symptoms were common in the 20 ME/CFS patients, such as unrefreshing sleep (19/20), post-exertional malaise (19/20), and impaired memory (also referred to by patients as **“**brain fog”, 19/20), similar to the overall cohort. The ME/CFS participants were separated into two groups (mild and severe), and samples were selected based on being at the extremes of mild or severe disease based on the MFI score. In the mild group the mean MFI-20 score was 58.4 (range 51-62) and in the severe group the mean MFI-20 score was 97.9 (range 96-100). The majority of the participants in both groups was female with a similar mean age (**Table 1**).

**Table 1:** Demographics of ME/CFS patients and control subjects.

	Severe ME/CFS (n = 10)	Mild ME/CFS (n = 10)	Controls (n = 20)	p value
MFI-20 score range (mean)	96-100 (97.9)	51-62 (58.4)	N/A	<0.0001
Age in years range (mean)	31-69 (54.3)	19-72 (50.1)	17-70 (51.2)	0.71
Sex male/female	1/9	2/8	4/16	0.77

N/A, Not applicable.

### EV Levels in ME/CFS Participants and Control Participants

**Figure 1:**
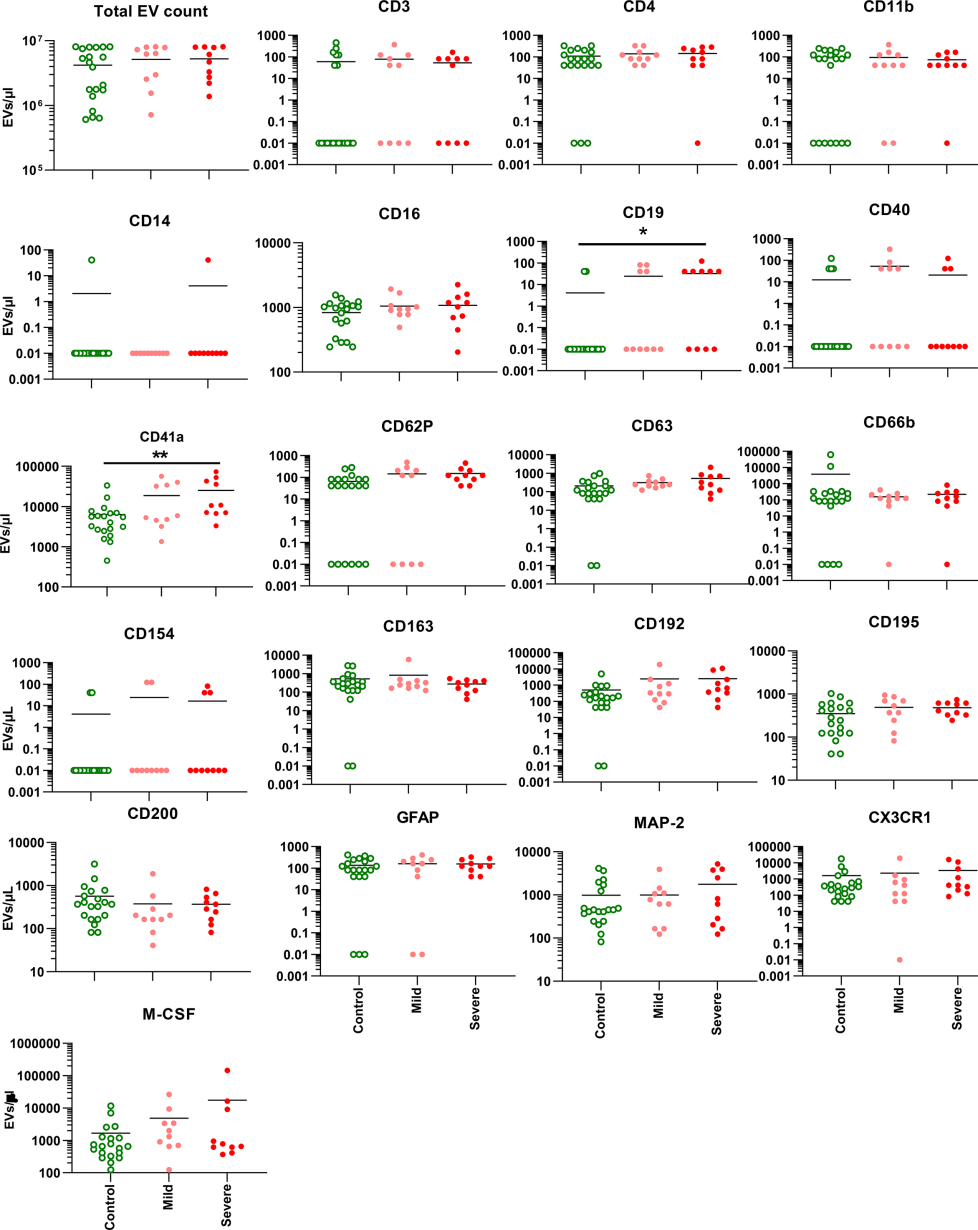
EV levels and phenotype. The concentration of EVs expressing a given cellular marker is shown for each of 20 markers, in addition to the total EV count. The bars represent mean EV levels for each group. Twenty control participants were compared to 10 each with mild or severe ME/CFS. The lower limit of EV detection was set as 0.01 EVs/μl. *p < 0.05, ** < 0.01 by ANOVA, with all groups compared to each other.

**Figure 2:**
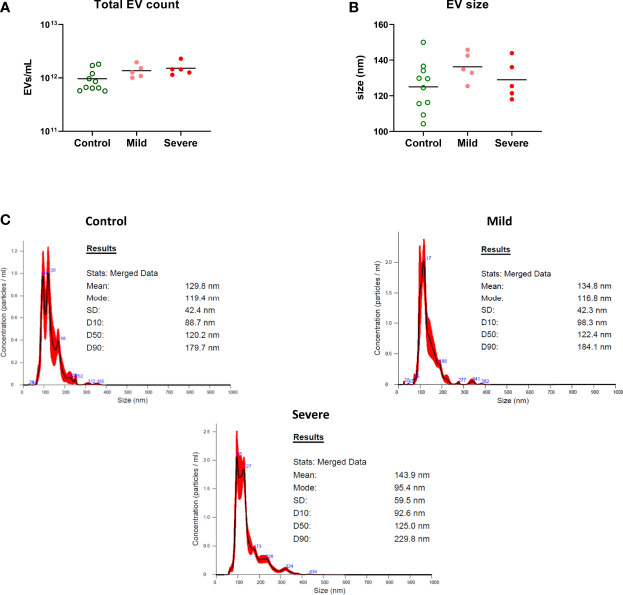
EV quantitation by nanoparticle tracking analysis. EV concentration was verified in a subset of 10 control participants and 5 each mild or severe ME/CFS participants. **(A)** EV particle counts and **(B)** the mean size distribution were determined by nanoparticle tracking analysis (NTA). There were no significant differences in total EV count and size characteristics of EVs between the groups by Kruskal-Wallis test and Dunn’s multiple comparison *post hoc* test. **(C)** Representative graphs of nanoparticle tracking analysis showing the EV particle-size distribution.

Overall EV levels measured by flow cytometry were compared between groups and were not found to differ based on ME/CFS status (**Figure 1**). A subset of half of the samples spanning the range of EV counts was also tested by nanoparticle tracking analysis, and again total EV counts were slightly but not significantly lower in healthy subjects compared to ME/CFS participants, and EV size also did not differ significantly between clinical groups (**Figure 2**). EVs bearing markers from a variety of cellular lineages were examined, including white blood cells (WBCs), platelets, and neuronal cells. The WBC markers included canonical T cell (CD3, CD4), B cell (CD19), and myeloid cell markers (CD11b, CD66b). Of these, CD19 was significantly higher in the severe ME/CFS participants compared to the healthy control participants (p=0.023, **Figure 1**). Of the platelet markers CD41a and CD62P, CD41a was also higher in the severe ME/CFS participants compared to the healthy control participants (p=0.015, **Figure 1**). None of the other markers including an exosome marker (CD63), cellular activation markers (CD40, CD154, CD163, CD192, CD195, M-CSF, CX3CR1), and neuronal markers (CD200, GFAP, MAP2) showed differences among study groups. Given the range age in the study population, and analysis of covariance (ANCOVA) was performed including age, and the associations between CD19+ and CD41a+ EVs and disease status remained significant. Of note, the differences seen between healthy control and severe ME/CFS participants would not have remained significant after correction for multiple comparisons.

## Discussion

In this study we compared EV levels among healthy control participants and those with mild vs. severe ME/CSF. This exploratory study was relatively small, with only 10 subjects in each of the ME/CFS disease categories. Modestly elevated levels of the B cell marker CD19 and the platelet marker CD41a were detected on EVs from participants with severe ME/CFS. While the changes did not remain significant after correction for multiple comparisons, the findings are nonetheless intriguing and worthy of further study in replication cohorts.

This ME/CFS population was part of a larger Stanford University cohort of patients with ME/CFS, where the severity of illness was based on MFI-20 and was correlated with cytokine levels. Seventeen of the 51 cytokine levels directly correlated with the severity of the illness in patients with ME/CFS (16). Prior studies on EV in ME/CFS, suggests that these vesicles could be a biomarker for this complex illness (7, 8, 10, 11). Several authors reported an increase in these EVs, and proteomic analysis could help identify a signature biomarker for this illness (7, 8, 10, 11). Of note, the two largest studies also were discrepant as to whether EV concentration differed in the plasma of healthy subjects compared to ME/CFS patients. Some of this variability may be due to the technique used to count EVs, which included nanoparticle tracking analysis (7, 10, 11) and flow cytometry (15). In the current study, EVs were quantitated by flow cytometry, and our finding of no difference in EV counts between clinical groups differed from the work of Eguchi et al. (8).

The novelty of our EV analysis compared with prior studies in ME/CFS patients was the identification of the cell phenotype of origin of circulating EVs (15, 17). Although we did not observe a difference in the levels of EV between ME/CFS participants by the severity of illness and the healthy controls, a significant difference was found in the EV cellular linage markers. Significant higher levels of the B cell marker CD19 and platelet marker CD41a were seen on EVs from the severe compared to mild ME/CFS cases (P < 0.05). Platelet derived EVs are the most abundant EV population found circulating in humans (18). Interestingly, these EVs have been shown by some groups to possess anti-inflammatory properties when taken up by myeloid or lymphoid cells, including induction of TGF-β secretion by myeloid cells, and are associated with low NK cell function (17, 18). Based on the prior abnormalities in certain cytokines levels found in this cohort, it is possible the activation of these two cell populations could be a consequence of the changes in cytokines. Low NK cell function is a well-documented and consistent finding in ME/CFS patient populations, as is elevated TGF-β, these findings offer a potential role for platelet derived EVs in the pathogenesis of ME/CFS (16, 19). From the published profile of seventeen abnormal cytokines in ME/CFS, IL-4, IL-7, and IFN-γ are involved with B cell development, maturation, and differentiation (20), and CXCL1 and CCL11 (eotaxin) are abundant in platelets (21–23).

In summary, using a flow cytometry platform to fingerprint EV count and phenotype, we found a signal that B cell and platelet derived EVs may be higher in ME/CFS patients, and this finding is worth exploring in replication cohorts. If the findings of elevated B cell or platelet-derived EVs were replicated in other studies it would allow study of potentially disrupted homeostatic pathways in ME/CFS pathogenesis.

## Data Availability Statement

The raw data supporting the conclusions of this article will be made available by the authors, without undue reservation.

## Ethics Statement

The studies involving human participants were reviewed and approved by Stanford University Institutional Review Board. The patients/participants provided their written informed consent to participate in this study.

## Author Contributions

HB, JM, and PN conceived of the study. HB and PN wrote the manuscript. DH, EM, and JA generated and analyzed data. All authors contributed to the article and approved the submitted version.

## Conflict of Interest

The authors declare that the research was conducted in the absence of any commercial or financial relationships that could be construed as a potential conflict of interest.

## Publisher’s Note

All claims expressed in this article are solely those of the authors and do not necessarily represent those of their affiliated organizations, or those of the publisher, the editors and the reviewers. Any product that may be evaluated in this article, or claim that may be made by its manufacturer, is not guaranteed or endorsed by the publisher.
