# Supplementary material for: Comparative Analysis of Extracellular Vesicles in Patients with Severe and Mild Myalgic Encephalomyelitis/Chronic Fatigue Syndrome
Source: Front Immunol. 2022 Mar 4;13:841910. doi: 10.3389/fimmu.2022.841910 (PMC8931328; doi:10.3389/fimmu.2022.841910)
Supplement: Supplementary file 1 [file Table_1.pdf]

Supplementary Table 1: Antibody characteristics

Marker	Fluorochrome	Clone	Isotype	µL per test	MFGR	Cat#
CD3	FITC	OKT3	Ms IgG2a, k	1	BioLegend	317306
CD4	PE	SK3	Ms IgG1, k	1	BioLegend	344606
CD14	APC	63D3	Ms IgG1, k	1	BioLegend	367118
CD16	V450	3G8	Ms IgG1, k	4	BD Biosciences	560474
CD19	PE-Cy7	SJ25CI	Ms IgG1, k	4	BD Biosciences	557835
CD66b	PerCP/Cy5.5	G10FS	Ms IgM, k	1	BioLegend	305108
CD41a	PerCP/Cy5.5	HIP8	Ms IgG1, k	1	BioLegend	303720
CD62P	FITC	AK-4	Ms IgG1, k	4	BD Biosciences	555523
CD63	APC	H5C6	Ms IgG1, k	1	BioLegend	353008
CD163	PE-Cy7	GHI/6I	Ms IgG1, k	1	BioLegend	333614
CD192	BV421	K036C2	Ms IgG2a, k	1	BioLegend	357210
CD195	PE	2D7CCR5	Ms IgG2a, k	4	BD Biosciences	555993
CD11b	PE-Cy7	ICRF44	Ms IgG1, k	1	BioLegend	301322
GFAP	BV421	2E1E9	Ms IgG2b	1	BioLegend	644710
MAP-2	Alexa 488	18MAP2B	Ms IgG1, k	4	BD Biosciences	560399
CD200	PerCP/Cy5.5	OX104	Ms IgG1, k	1	BioLegend	329216
CD40	FITC	5C3	Ms IgG1, k	1	BioLegend	555588
CD154	APC	2431	Ms IgG1, k	1	BioLegend	310810
CX3CR1	BV421	2A9-1	Rat IgG2b, k	1	BioLegend	341620
M-CSF	PE	26786	Ms IgG2a	1	R&D Systems	IC2161P
